# miRNA accumulation correlates with increased phloem cell proliferation in tomato *hawaiian skirt* mutants

**DOI:** 10.3389/fpls.2025.1649913

**Published:** 2025-09-16

**Authors:** Fabien Lombardo, Tarek El mestari, Thomas Kirat, Muhammad Ijaaz Jondah, Julie Marsaudon, Norbert Bollier, Chiaki Mastukura

**Affiliations:** ^1^ Faculty of Life and Environmental Sciences, University of Tsukuba, Tsukuba, Japan; ^2^ Université de Bordeaux, Collège Sciences et Technologies, Talence, France; ^3^ Université de Bordeaux, INRAE, UMR1332 Biologie du Fruit et Pathologie, Villenave d’Ornon, France

**Keywords:** phloem, tomato, pedicel, cambium, radial growth

## Abstract

**Introduction:**

The cambium is the meristem responsible for production of plant vascular tissues, the xylem (wood) and phloem, during plant radial growth. Due to its internal location and relatively low cell number, the biology of the cambium remains less well understood compared to other plant meristems. The tomato *hws* mutant exhibits miRNA accumulation and produces a markedly enlarged phloem, suggesting a role for miRNAs in regulating vascular development. This study aims to identify the accumulated miRNAs associated with the vascular phenotype in *hws* mutants and characterize their impact on phloem development kinetics.

**Methods:**

To achieve this, we analyzed the vascular phenotype of *hws* mutants using pedicel and stem sections examined using light and scanning electron microscopy, alongside RNA sequencing of miRNAs from pedicels.

**Results:**

Our findings reveal increased cell division rates during the phloem expansion phase in *hws-1* while xylem is reduced due to a shortened expansion phase in pedicels. Key miRNAs, including miR319 and miR164, were identified as highly accumulated in the mutant pedicels. Vascular phenotypes in stems were consistent with those observed in pedicels.

**Discussion:**

Our findings underscore the potential for manipulating miRNA levels to improve phloem capacity and possibly crop yields through targeted genetic strategies.

## 1 Introduction

The emergence of vascular tissues marked a key step in plant evolution (Lucas et al., 2013). This innovation enabled efficient transport of water and nutrients, allowing plants to colonize land and grow to considerable heights. Vascular tissues consist mainly of xylem and phloem, both originating from the same meristem (Fukuda and Ohashi-Ito, 2019). Understanding the biology of the vascular meristem is not only of scientific interest but also has practical implications: xylem forms wood, a major commodity, while phloem is essential for sugar distribution within plants, impacting crop productivity. Several theoretical models supported by multiple studies suggest that photoassimilate translocation from source to sink organs is a major bottleneck in yield improvement (Konrad and Roth-Nebelsick, 2023; Patrick, 2013). For example, in giant pumpkins, increased phloem size correlates with the enormous fruits produced (Savage et al., 2015). Similarly, in tomato (*Solanum lycopersicum*), a comparative study of vascular tissue across cultivars revealed a strong correlation between pedicel phloem size and fruit size (Simon et al., 2022). More direct evidence comes from Nam et al., 2022, who achieved a 60% increase in total fruit weight by knocking down *SlJULGI*, a negative regulator of phloem development.

Tomato has bicollateral vascular bundles, with both an inner and an outer phloem flanking the xylem. Anatomical studies of species that form bicollateral bundles show that the outer phloem most likely originates from the procambium, whereas the cambium produces the secondary inner phloem and secondary xylem (Beck, 2010; Esau, 1977). Research on the biology and functional differences between the inner and outer phloem is scarce, but studies in Cucurbitaceae have shown that the outer phloem preferentially expresses genes involved in processes other than sugar transport, including stress responses, defense, ion transport, and secondary metabolite biosynthesis (Sui et al., 2018; Sui et al., 2021).

Previously, we showed that the inner pedicel phloem of the *hawaiian skirt-1 (hws-1)* tomato mutant is at least twice as large as that of the wild type (Lombardo et al., 2021a). This enlargement is associated with increased pedicel girth, suggesting enhanced division of phloem progenitor cells in the mutant. Although the size and number of fruits remain unchanged in the mutant, their Brix is higher, a difference attributed to enhanced sugar translocation from the enlarged phloem. The *HWS* gene emerges as a promising genetic target for engineering crops with enlarged phloem to improve yields.

Evidence from Arabidopsis, poplar, and tomato points to *HWS* as a regulator of microRNA (miRNA) biosynthesis (Damayanti et al., 2019; Dash et al., 2015; Lang et al., 2018; Mei et al., 2019; Zhang et al., 2017). The phenotypic traits in *hws* mutants are attributed to the accumulation of miRNAs and the resulting downregulation of their target genes. Since *HWS* encodes an F-box protein, it is thought to mediate the ubiquitin-dependent degradation of one or more target proteins. Recent preprint data from Gonzalo et al., 2024 suggest that MED37, a component of the MEDIATOR complex, is a direct target of HWS. In *hws* mutants, stabilization of MED37 would promote the recruitment of MIRNA loci to the nuclear pore complex. This would lead to enhanced co-transcriptional processing of pri-miRNAs and increased production of mature miRNAs. Additionally, miRNA loading onto ARGONAUTE would be impaired, resulting in an increase of unloaded miRNAs free to move between cells. Thus, reduced HWS activity would correlate with both elevated miRNAs levels and greater non-cell-autonomous miRNA movement.

The miRNA miR165 and miR166 are known to regulate HD-ZIP III transcription factors, which are necessary for proper patterning of vascular tissues (Ramachandran et al., 2017). However, there is currently no evidence directly linking miRNA activity to the promotion of phloem development. Our earlier work showing expanded pedicel size in *hws* mutants suggests that miRNAs may promote radial growth, though the identity of the recipient cells and timing remains unclear.

The cambium, the meristematic tissue responsible for the production of vascular tissues during radial growth, is less well understood than apical meristems. A study in Arabidopsis reports that phloem and xylem are derived from a single stem cell lineage, termed bifacial cells (Shi et al., 2019). In contrast, studies in Populus identify two types of stem cells, indicating that the identity of cambial stem cells may vary among the species (Bossinger and Spokevicius, 2018; Du et al., 2023). In tomato, no studies have yet examined whether cambial stem cells are bifacial. Thepositioningofauxinsignalingmaximadeterminewhethercambialcellsdifferentiate into xylem or phloem (Smetana et al., 2019). Gibberellins direct auxin flow and thereby influence the xylem-to-phloem ratio (Mäkilä et al., 2023). In apical meristems, stem cells give rise to transient amplifying (TA) cells that rapidly proliferate before undergoing terminal differentiation. These cells have lost their pluripotency but are not yet fully differentiated, serving as intermediate population between stem cells and mature tissue. In the cambium, cell divisions are mostly observed in stem cells (Shi et al., 2019; Smetana et al., 2019) and the lack of clearly defined transient zone raises questions about the applicability of the TA cell concept (Hunziker and Greb, 2024), although other studies assert unequivocally their existence (Fischer et al., 2019).

To investigate how the *hws* mutation affects phloem proliferation and identify specific miRNAs that accumulate in the mutant vasculature, we analyzed histological sections of pedicels and stems, and quantified miRNAs from pedicel tissues. Our findings suggest altered developmental timing in the mutant, and confirm a role for *HWS* in cambium regulation.

## 2 Materials and methods

### 2.1 Plant material

The tomato (*Solanum lycopersicum*) *hws-1* and *hws-3* lines were isolated from an ethyl methanesulfonate (EMS) mutagenized population of the “Micro-Tom” cultivar with the identifiers TOMJPE8986 and TOMJPW3299, respectively (Saito et al., 2011). Description of the corresponding mutations in *HAWAIIAN SKIRT (Solyc01g095370)* can be found in Damayanti et al., 2019.

All experiments were performed on BC3 (*hws-1*) and BC1 (*hws-3*) populations grown in rockwool supplemented with a Ōtsuka house 1 and 2 (OAT Agrio Co., Ltd, Tokyo, Japan) nutrient solution under a 16-h light/8-h dark regime provided by LED lights with an output of 230 µmolm⁻²s⁻¹ of PAR.

### 2.2 Microscopy and vascular tissue growth kinetics

Pedicels were cut off plants and immediately sectioned using a VT1200S vibratome (Leica Microsystems, Wetzlar, Germany) at a thickness of 60µm about halfway between the abscission zone and the fruit, which corresponds to the region of smallest diameter. For light microscopy, sections were directly stained with 0.05% toluidine blue for approximately 1.5 minute, briefly rinsed with distilled water and observed under an Olympus BX53 light microscope (Olympus Corporation, Tokyo, Japan) using an imaging software (cellSens Standard 1.6, Olympus, http://www.olympus-global.com; accessed on 14 March 2024). A minimum of five pedicels from three different plants were observed for each timepoint in the kinetics experiment. The area of vascular tissue was measured manually in QuPath (Bankhead et al., 2017) using the wand tool. Automatic segmentation was not used because it did not reliably delineate tissue boundaries in these samples. Tissues were identified based on toluidine blue staining intensity and hue, anatomical location, and general cellular morphology, which were more consistently distinguished by eye.

Stems of three-month-old plants were sectioned at the middle of the fourth internode and stained as described above for observation under an Olympus SZX10 stereo microscope (Olympus Corporation, Tokyo, Japan).

Because the pedicel tissues displayed successive phases with approximately linear kinetics, separated by shifts in growth rate, we analyzed growth kinetics using segmented (piecewise) linear regression rather than a logistic (sigmoid) model. Candidate breakpoints were first identified by visual inspection of the growth trajectory (i.e. DAA = 8 and 14 for WT; 6 and 14 for *hws-1*). These values were then provided as initial estimates to the segmented package in R, which optimized breakpoint positions to obtain the best-fitting continuous piecewise model (Muggeo, 2003; Wickham, 2016).

For scanning electron microscopy, pedicel sections were prepared following the protocol of (Mullendore et al., 2010) with the following modifications: Pedicel segments from WT and mutant lines cut between the abscission zone and the calyx were directly immersed into superchilled 100% ethanol and stored overnight in a −20 °C freezer. In the proteinase K solution, calcium acetate was replaced with calcium chloride and a 5% Triton X-100 was used. Proteinase K incubation time was reduced to five days. Prior to observation with a JSM-IT500HR electron microscope (Jeol Ltd., Tokyo, Japan), samples were dehydrated, freeze-dried and coated with 1nm platinum/palladium. Images were edited for brightness, sharpness and color balance for consistency across images using Adobe Photoshop (Adode Inc., San Jose, CA, USA). For the phloem cell size measurement, images were first segmented using Cellpose 2.0 (Stringer et al., 2021) and the trained model TN3, then, the segmented objects were manually curated to only keep the cells of the phloem tissue (sieve tubes, companion cells, parenchyma cells and sclereids) and the cell area was measured using ImageJ/Fiji (Schindelin et al., 2012).

### 2.3 RNA sequencing and analysis

The distal parts of pedicels, from above the abscission zone to below the calyx, of 10-DAA WT and *hws-1* fruits were frozen into liquid nitrogen. Five pedicel segments were bulked into one sample to obtain about 50mg of material and ground in a mortar using a pestle. Total RNA was extracted using a High Pure RNA Isolation kit (Roche, Basel, Switzerland) following manufacturer’s instructions (single-column protocol). Three biological replicates for each line were sequenced using a Illumina NovaSeq X Plus platform at a depth of one million-read per sample, which was estimated sufficient to capture the most abundand miRNAs, by Rhelixa Inc. (Tokyo, Japan).

Raw sequence data was analyzed with the sRNAminer software using the One Step pipeline with default settings (Li et al., 2024).

## 3 Results

### 3.1 The degree of phloem enlargement is dependent on *hws* allele severity

To date, the vascular phenotype of the tomato *hws* mutant had only been investigated in the weak allele *hws-1* (Lombardo et al., 2021a). Previous studies in Arabidopsis showed that miRNA accumulation in *hws* lines is dependent on the severity of the allele (Lang et al., 2018; Mei et al., 2019). The *hws-3* allele introduces a premature stop codon near the middle of the coding sequence, resulting in pronounced leaf deformations and severely reduced seed production, and is therefore considered a strong allele (Damayanti et al., 2019; **Supplementary Figure S1**). Based on these observations, we hypothesized that *hws-3* would display enhanced miRNA accumulation and, consequently, a greater degree of phloem enlargement.

**Figure 1:**
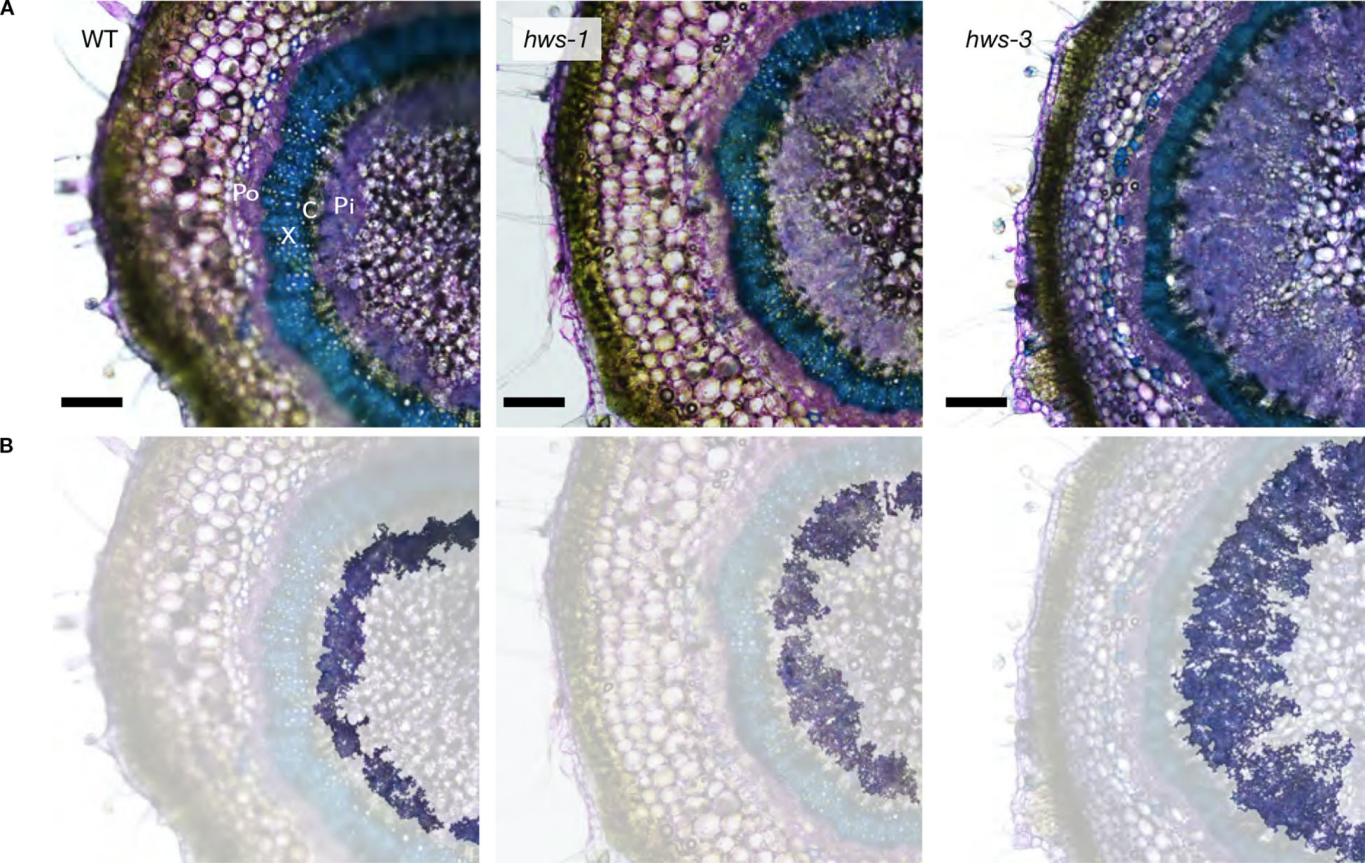
Pedicel cross-sections of WT, *hws-1* and *hws-3* show progressive increases in phloem area. **(A)** Section were stained with toluidine blue staining to visualize xylem (X; in blue) and outer (Po) and inner phloem (Pi; in purple); cambium (C). Bars indicate 200 µm. **(B)** Same images as in **(A)**, with other tissues masked to highlight the inner phloem.

To compare vascular phenotypes between *hws-1*, *hws-3* and WT plants, we analyzed sections of pedicels at 20 DAA. Sections were stained with toluidine blue to visualize phloem and xylem tissues under the microscope. As previously reported in an earlier study, the inner phloem of *hws-1* was markedly enlarged, while the outer phloem was unaffected (**Figure 1**). We measured outer phloem area and confirmed it was unchanged across genotypes (**Supplementary Table S1**). Because the outer phloem is not derived from the cambium, represents only a small fraction of the pedicel area and shows no genotype-specific difference, we excluded it from further analysis. Hereafter, we refer to the inner phloem simply as “phloem” for brevity. In the severe *hws-3* allele, the phloem was strikingly enlarged, confirming our expectations. Both mutant lines showed narrower xylem compared to WT, with this effect appearing more pronounced in *hws-3*.

**Figure 2:**
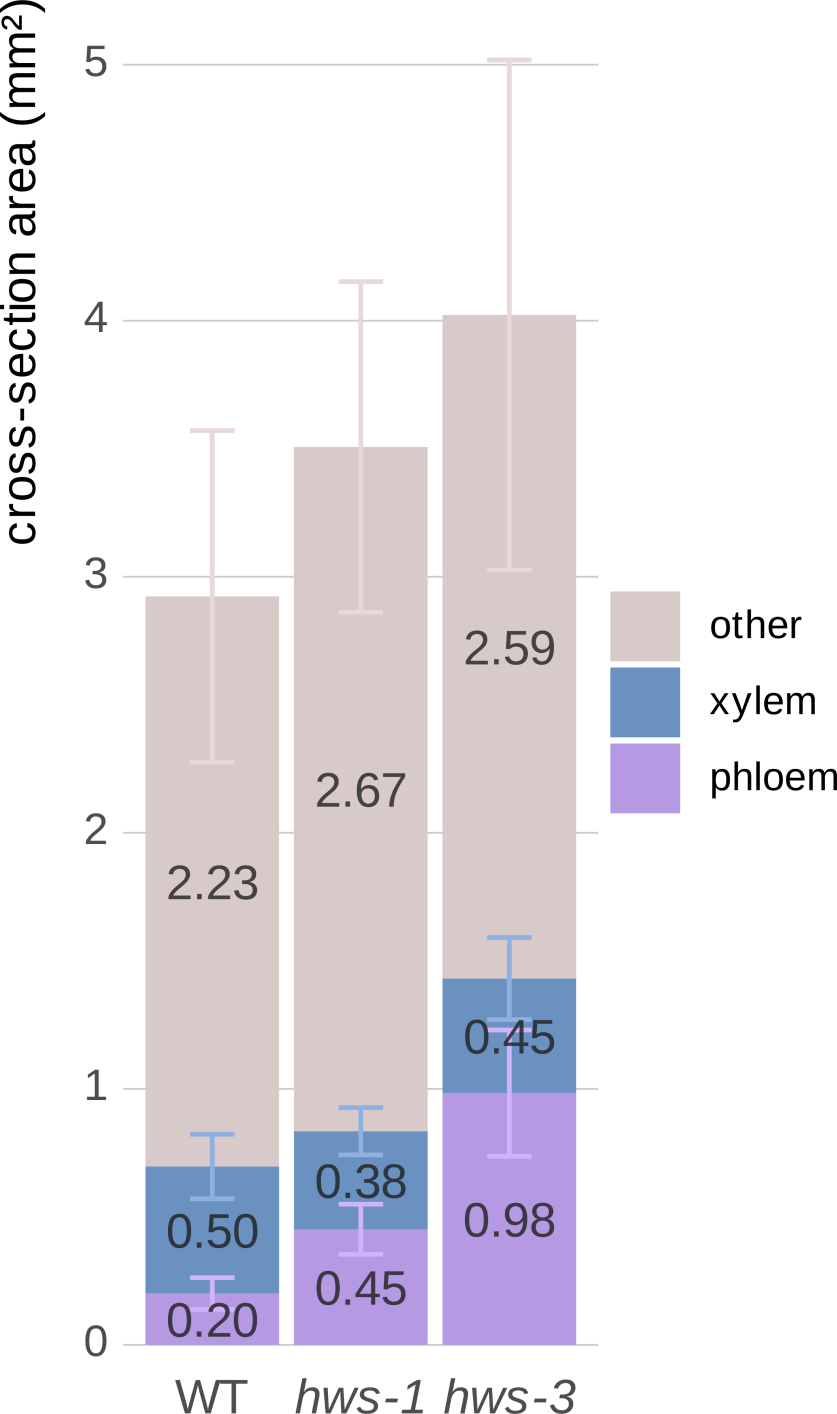
Quantitative comparison of phloem and xylem tissue cross-sectional areas in 20-DAA pedicels from WT, *hws-1* and *hws-3*.

To quantify vascular tissue changes, we manually delineated section images of 20-DAA pedicels using the QuPath segmentation software (Bankhead et al., 2017). Mutant pedicels were larger, showing a 20% and close to a 40% increase in total cross-sectional area for *hws-1* and *hws-3*, respectively (**Figure 2**). Phloem area was more than twice as large in *hws-1*, with an average increase of 125%, slightly exceeding our initial estimate (Lombardo et al., 2021a). The *hws-3* line exhibited a phloem enlargement nearly fivefold relative to WT, confirming the correlation between allele severity and phloem expansion.

In contrast, xylem area reduction was not strictly correlated with allele strength. In the strong *hws-3* line, the xylem showed a less pronounced reduction than expected, counterintuitive to our visual inspection of the sections. As pedicel expands, the xylem is pushed outward, increasing its diameter. In mutant pedicels, the xylem tissue is distributed over a broader circumference, which partially explains the relatively modest reduction in total area compared to their visibly narrower morphology. Thus, despite appearing thinner in section, the total xylem area does not decrease proportionally with phloem expansion.

Non-phloem and non-xylem tissues in *hws-1* and *hws-3* showed an approximate 15% to 20% increase, mostly from the enlargement of the central parenchyma, as evidenced by microscopy (**Figure 1**).

These observations were confirmed statistically using non-parametric tests, as normality could not be assessed for *hws-3* due to its small sample size (*n* = 5). For each tissue type, differences among lines were assessed with the Kruskal–Wallis test, followed by pairwise Wilcoxon rank-sum tests when significant. Holm’s correction was applied for multiple comparisons. Phloem area differed strongly among lines (*p* = 4.3 × 10^−8^), with all pairwise contrasts remaining significant after correction (WT vs *hws-1*, *p* = 1.2 × 10^−8^; WT vs *hws-3*, *p* = 9.4 × 10^−5^; *hws-1* vs *hws-3*, *p* = 1.2 × 10^−4^). Xylem (*p* = 0.052) and other tissues (*p* = 0.066) showed no significant differences.

Area mean values are indicated on each corresponding bar. All remaining tissues, apart from xylem and phloem, are grouped as “other”. Error bars show mean average deviations. *n* = 19, *n* = 24 and *n* = 5 for WT, *hws-1* and *hws-3*, respectively. Phloem area significantly differed among lines (all pairwise *p* < 0.001); xylem and other tissues were not significantly different.

### 3.2 Phloem cell size distribution is comparable across WT and *hws* lines

**Figure 3:**
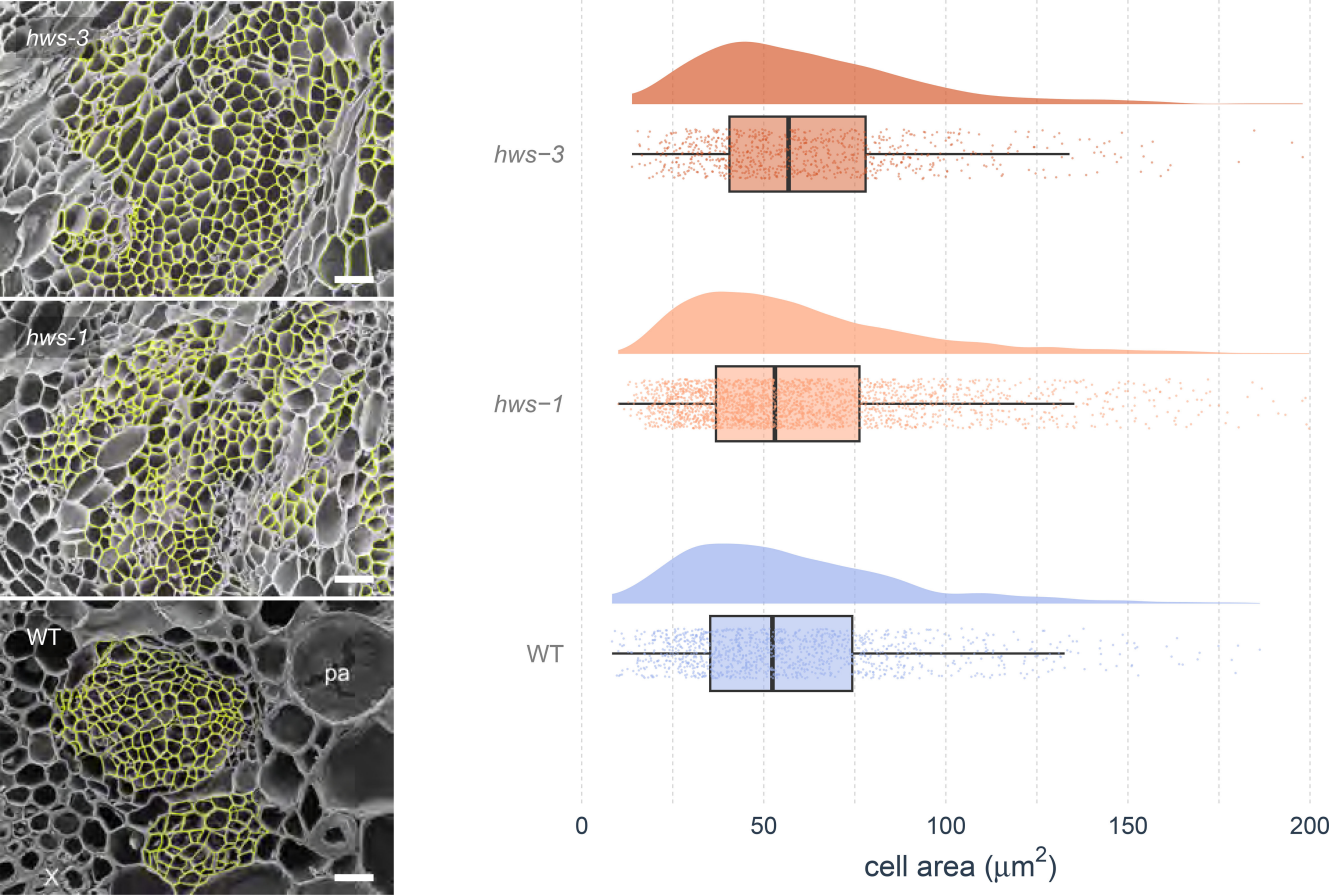
Analysis of phloem cell sizes in WT and *hws* mutant lines.

To determine whether phloem enlargement in *hws* mutants arises from increased cell size or cell number, we conducted morphological analyses using scanning electron microscopy (SEM) and automated image segmentation. Phloem cell sizes were quantified using the Cellpose segmentation software (Stringer et al., 2021). Density plots of cell sizes were comparable across all genotypes, with a median value of approximately 60 µm (**Figure 3**). Cell size distributions did not differ significantly among lines in Bonferroni-corrected pairwise Anderson–Darling tests (all adjusted *p* = 1), and Wasserstein distances ranged from 2.62 to 4.34, consistent with the similar density plots. SEM analysis revealed no discernible structural differences in phloem morphology between mutant and WT plants, beyond the observed overall size increase. These findings indicate that the enlarged phloem area in *hws* mutants arises from an increase in cell number rather than changes in cell sizes, with structural characteristics were preserved relative to WT.

Left: Scanning Electron Microscopy (SEM) images of 20-DAA pedicel cross-sections. Phloem cells (highlighted in yellow) were segmented using the Cellpose software before manual curation. Bars indicate 25 µm. Right: Distributions of cell sizes measured from SEM images using the Cellpose software showing comparable cell densities across genotypes, with no statistical differences (pairwise Anderson–Darling tests, Bonferroni-corrected, all adjusted *p* = 1; Wasserstein distances show small differences. Half-eye plots show cell area distributions. Boxes indicate the interquartile range and median; points represent individual values; cloud width reflects data density. *n* = 3, 6 and 7 for *hws-3*, *hws-1* and WT, respectively).

### 3.3 Vascular development timing and phloem expansion rates shift in *hws-1*

To investigate the kinetics of phloem development, we measured vascular tissue area over a 40-day period beginning at anthesis. Because the *hws-3* mutant produces very few seeds, we focused our analysis on the *hws-1* allele, which is more amenable to experimental investigation.

**Figure 4:**
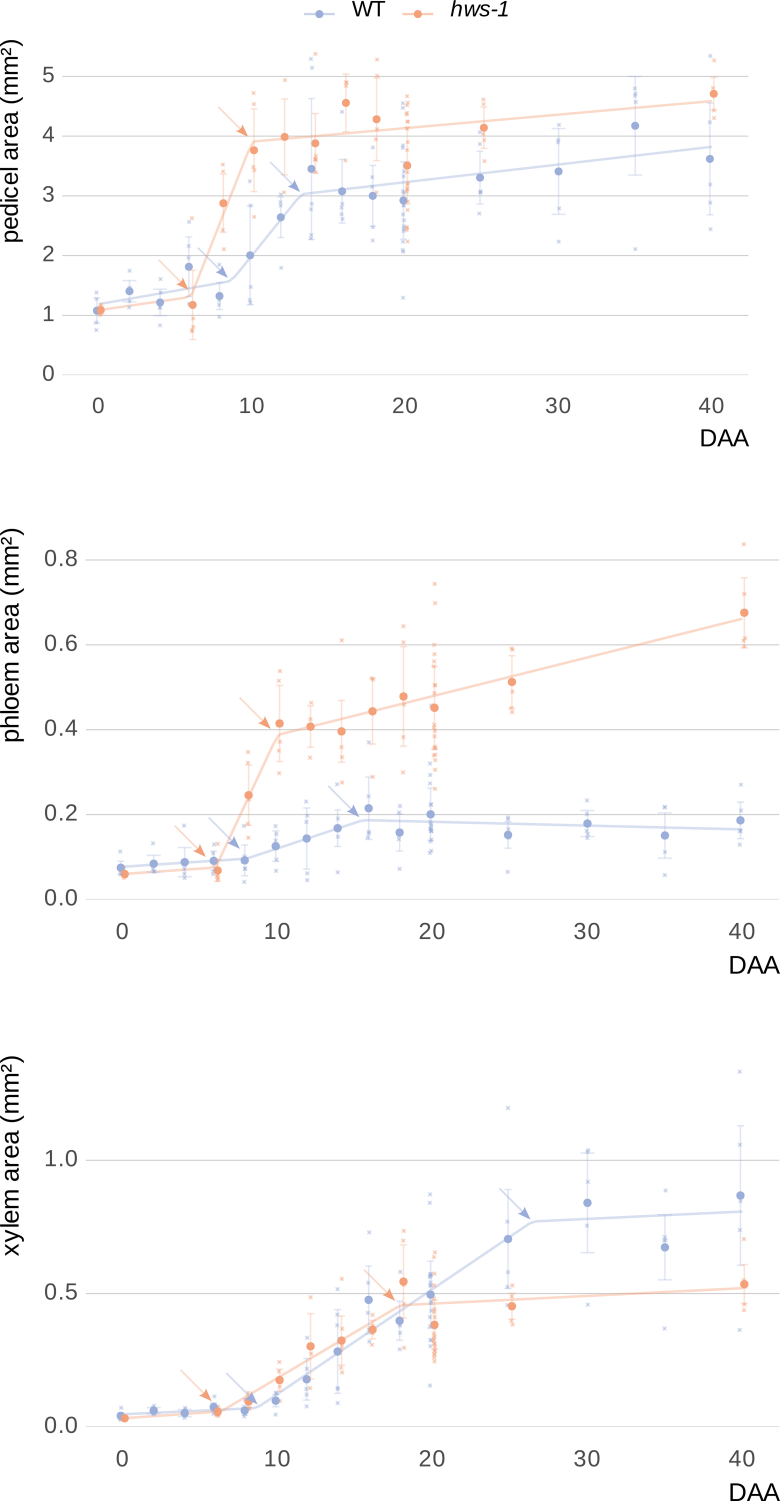
Kinetic analysis of vascular tissue expansion during pedicel development in WT and *hws-1*.

Pedicels were sectioned and stained with toluidine blue following the same methodology as in our earlier experiment for manual tissue delineation. Plotting cross-sectional areas of the whole pedicel, phloem and xylem over time revealed that growth in each followed three distinct phases across all genotypes (**Figure 4**).

During the early phase, both phloem and xylem showed minimal expansion, likely progressing at rates comparable to those of parenchymatic tissues. In *hws-1*, this early phase ended around the 6-DAA mark, approximately two days earlier than in the WT line. This was followed by a period of rapid expansion, during which phloem area increased significantly faster in the mutant, at an approximate rate of 0.08mm²d⁻¹. This rate was more than six times higher than in the WT; however the duration of the phloem rapid phase, only about four days in the mutant, was significantly shorter than in the WT, where it lasted nearly eight days (**Table 1**). In the late phase, WT phloem ceased growing entirely, whereas *hws-1* phloem continued to expand at a slow but sustained rate.

**Table 1:** Comparison of phloem and xylem expansion rates and phase durations (days) during pedicel development.

Tissue	Line	Early phase	Rapid phase	Late phase
phloem	WT	0.002 (8 d)	0.012 (7.5 d)	-0.001
	*hws-1*	0.003 (6 d)	0.078 (4 d)	0.009
xylem	WT	0.003 (8.7 d)	0.039 (17.7 d)	0.003
	*hws-1*	0.004 (6.4 d)	0.034 (11.5 d)	0.003

Expansion rates (in mm²d⁻¹) correspond to slopes from correlation curves in **Figure 4**. Values highlight a markedly faster phloem development as well as a shortened rapid phase in the mutant.

In contrast to the substantial differences in phloem expansion kinetics, xylem growth rates were nearly identical between lines for each of the three phases (**Table 1**). However, the duration of the xylem rapid phase was considerably shorter in *hws-1*, lasting less than twelve days, that is approximately six days shorter than in the WT line.

Pedicels were analyzed over forty days post-anthesis, revealing three distinct growth phases: an early phase with minimal expansion, a rapid expansion phase, and a late phase. Phloem expanded significantly faster in the mutant. While xylem growth rates were similar between WT and mutant, the duration of both phloem and xylem rapid phases were shorter in *hws-1*, indicating altered vascular developmental timing. Each dot represents the average of at least five sections from pedicels of at least three different plants (91 and 71 pedicels in total for WT and *hws-1*, respectively). Arrows indicate breakpoints between different growth phases. Bars show median absolute deviations.

### 3.4 Stem radial growth is affected by the *hws* mutation

Previously, two-month-old *hws-1* plants were reported to exhibit a stem diameter approximately 1mm larger than that of WT controls (Damayanti et al., 2019); however, the vascular phenotype associated with this increase had not been characterized. To assess whether the large-phloem phenotype observed in pedicels also occurs in the stem, we conducted histological analysis of stem cross-sections using light microscopy.

**Figure 5:**
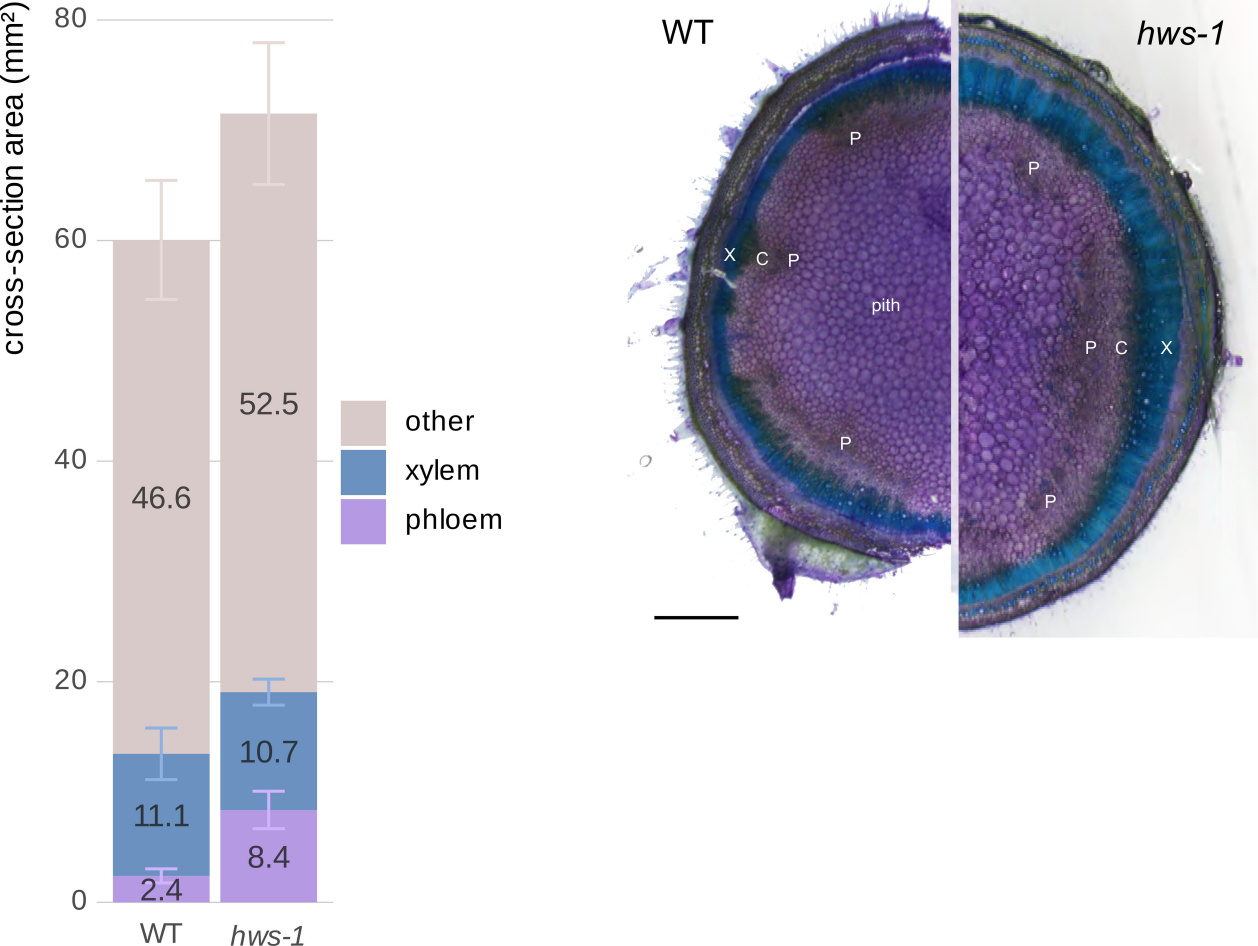
Comparison of phloem and xylem cross-sectional areas in three-month-old WT and *hws-1* stems.

To ensure that radial growth was complete and tissues were fully developed, we analyzed tissue areas in three-month-old plants. Consistent with our findings in pedicels, *hws-1* mutant stems showed a 3.5-fold increase in phloem cross-sectional area compared to WT controls (**Figure 5**). Tissue differences were assessed using the same non-parametric approach as for pedicels. Phloem area differed significantly among genotypes after Holm-corrected Wilcoxon tests (*p* = 5.7 × 10^−6^). The total area of tissues other than xylem and phloem also differed significantly (*p* = 0.0290). *hws-1* stems further showed a trend toward reduced xylem and increased parenchymatic tissue, although the xylem difference was not significant (*p* = 0.622).

These findings demonstrate that the influence of the *hws* mutation is not restricted to pedicel vascular tissue but extends to the stem, indicating a consistent effect on vascular development during radial growth.

Left: The *hws-1* mutation causes a 3.5-fold increase in phloem area compared to WT, accompanied by reduced xylem area and expanded parenchymatic tissues. This pattern mirrors the vascular phenotype observed in pedicels. Phloem differed significantly between genotypes (*p* < 0.001), as other tissues (*p* < 0.05), while xylem did not (*p* = 0.622). Area mean values are indicated on each corresponding bar. Error bars show mean average deviations. *n* = 11 and *n* = 10 for WT and *hws-1*, respectively. Right: Cross-section showing larger phloem (P) in *hws-1*. X: xylem, C: cambium. Scale bar represents 1 mm.

### 3.5 Identification of accumulated miRNA species in *hws* pedicels

Our central hypothesis posits that the miRNA accumulation in *hws* mutants leads to the repression of key genes involved in vascular tissue regulation. To identify these genes, we extracted total RNA from 10-DAA pedicels, which are expected to be the rapid expansion phase, from WT and *hws-1* plants and sequenced small RNAs.

Raw sequencing data were analyzed using the sRNAminer pipeline (Li et al., 2024), and revealed nine miRNAs with an enrichment of more than 1.5-fold compared to the WT controls. Notably, miR319b showed a very large increase (about 40-fold, *p* = 0.02), while miR164b showed a 2.6-fold increase (*p* = 0.12) (**Table 2**). Both miRNAs have been previously reported to be enriched in Arabidopsis *hws* mutants (Lang et al., 2018; Mei et al., 2019), with miR164 specifically linked to the altered leaf and floral phenotypes (González-Carranza et al., 2017). Some comparisons did not reach statistical significance, which we attribute to variability in pedicel size even at the fixed time point of 10 DAA, as previously observed (Lombardo et al., 2021a), and to the use of whole pedicel sections for RNA extraction, both of which increase variance across samples. Nevertheless, we believe that the consistent fold-change patterns support the same biological interpretation.

**Table 2:** Accumulated miRNAs in pedicels of WT and *hws-1*.

			TPM		
miRNA designation	Sequence	Length	WT	*hws-1*	Fold change
miR319b-Probable-3p-mature	TTGGACTGAAGGGAGCTCCT	20	369.7 (33.9)	14943.5 (2594.2)	40.4 (0.02)
miR164b-Known-5p-mature	TGGAGAAGCAGGGCACGTGCA	21	372.2 (57.6)	960.7 (273.3)	2.6 (0.12)
miR319c-Probable-3p-mature	TTGGACTGAAGGGAGCTCCCT	21	98.6 (11.9)	243.3 (30.3)	2.5 (0.02)
miR5300-Known-3p-star	TGGTATGCTTTGATTGGGAAAG	22	51.1 (8.5)	108.0 (38.7)	2.1 (0.18)
miR396c-Known-3p-star	GTTCAAGAAAGTTGTGGGAAA	21	116.7 (19.9)	243.1 (23.4)	2.1 (0.01)
miR164b-Known-3p-star	CATGTGCCTGTTTTCCCCATC	21	101.5 (41.9)	203.2 (31.9)	2 (0.08)
miR171b-Known-5p-mature	TATTGGCCTGGTTCACTCAGA	21	60.0 (10.2)	97.7 (7.8)	1.6 (0.03)
miR394b-Known-3p-star	AGGTGGGCATACTGTCAACAG	21	14.7 (1.4)	23.9 (8.5)	1.6 (0.32)
miR6027-Known-3p-star	TGAATCCTTCGGCTATCCATA	21	612.9 (59.4)	917.4 (119.5)	1.5 (0.07)

Transcripts per million (TPM) values represent averages of three samples. Mean absolute deviations and Welch’s *t*-test *p*-values are indicated in parentheses for TPM and fold change values, respectively. “Probable” miRNAs are miRNAs predicted based on computational criteria and evidence from sequencing data but lack experimental validation.

Left: one-month-old plants showing increasing leaf deformations across lines. Bars indicate 1cm. Right: positions of the mutations in *hws-1* and *hws-3*; modified from Damayanti et al., 2019.

Putative and demonstrated tomato gene orthologs of major *A. thaliana* genes associated with cambium size or differentiation are listed.

## 4 Discussion

In this study, we demonstrated that the strong phloem enlargement in tomato *hws* mutants results from an increase in cell division of phloem progenitor cells. The severe *hws-3* allele showed an even greater phloem expansion than *hws-1*, correlating the effect on phloem tissue with the loss of function of *HWS*. We also showed that the timing of vascular tissue development is altered in *hws-1*, further suggesting a role for miRNAs in coordinating this process. Finally, we identified specific miRNAs, such as miR319 and miR164, that accumulate strongly in the mutant, suggesting a regulatory role for these miRNAs in radial growth.

In a previous study, we reported that the *hws-1* mutant developed a markedly enlarged phloem, which had implications for sugar transport and yield (Lombardo et al., 2021a). There are two key areas of interest in controlling phloem development: first, from an agricultural perspective, plants with larger phloem are expected to exhibit improved yields due to increased bulk flow and more efficient phloem unloading (Patrick, 2013). This hypothesis is supported by a study in tomato demonstrating that suppression of *JULGI (JUL)*, a negative regulator of phloem development, results in significantly higher fruit yield (Nam et al., 2022). Secondly, generating plants with varying degrees of phloem enlargement would provide experimental material to investigate the physical mechanisms underlying phloem function. Such material is scarce, and our current understanding of phloem function remains largely theoretical due to limited empirical evidence (Konrad and Roth-Nebelsick, 2023).

The HWS protein belongs to the F-box family, whose members are known to ubiquitinate target proteins for subsequent degradation by the 26S proteasome. Phenotypes of *hws* mutants are consequently expected to result from the accumulation of one or several proteins involved in miRNA biosynthesis. However, the actual targets of HWS have long remained elusive. A recent preprint study reported the identification of subunits of the nuclear pore-located MEDIATOR complex as targets of HWS, directly linking miRNA accumulation in *hws* mutants to enhanced co-transcriptional processing of miRNAs (Gonzalo et al., 2024). This is consistent with studies in Arabidopsis showing a negative correlation between miRNA levels and HWS activity (Lang et al., 2018; Mei et al., 2019).

In this study, we show that the severe *hws-3* allele, believed to represent a complete *HWS* loss of function, exhibits a phloem about twice as large as that of *hws-1*. Given the strong functional conservation of *HWS* across species (Lombardo et al., 2021b), we anticipate that manipulating the *HWS* gene would influence miRNA levels and enable the generation of plants with larger phloem tissues and associated improved agricultural yields.

In our earlier work, under the assumption that tomato cambial cells are bifacial, we questioned whether the increase in phloem area resulted from an imbalance in the fate decision mechanism favoring the phloem fate in the mutant (Lombardo et al., 2021a). Here, we observed a comparable reduction in xylem area between both weak and severe *hws* alleles, indicating that the phloem does not develop at the expense of the xylem. Our microscopy analysis of pedicels confirmed that the increase in phloem size was not the result of enlarged cells but the consequence of a higher rate of cell division during a rapid expansion phase. To the best of our knowledge, this phenotype is unique among vascular mutants described in the literature. Although the tomato *Sljul* mutant produces an enlarged phloem evocative of the phenotype of *hws-1*, the diameter of *jul* peduncles is similar to that of WT controls, indicating that the phloem enlargement occurs at the expense of surrounding tissues. The *jul* mutation de-represses the *SlSMXL5* gene, which in turn promotes phloem differentiation. The *hws* mutation, in contrast, promotes the division of phloem progenitor cells by yet unclear mechanisms. The identity of the vascular cells whose division is promoted by loss of *HWS* function remains unclear. In *hws* mutants, we did not observe a marked increase in the area of the central cambial region or enhanced xylem expansion. Previous work has shown that mutations in *HWS* can rescue the short-root phenotype of the Arabidopsis *shortroot* mutant by promoting TA cell division (Kim et al., 2016). However, the concept of TA cells, well defined in apical meristems, is challenging to apply to the cambium due to the limited number of cells and absence of positional markers for their identification (Wybouw et al., 2024). Reporter gene fusion experiments indicate that *HWS* is expressed in the phloem-pole pericycle (Kim et al., 2016), suggesting that *hws* mutations may influence cells outside the central cambium. In the model proposed by Gonzalo et al., 2024, impairment of *HWS* function leads to both increased miRNA abundance and enhanced cell-to-cell mobility of miRNAs. Except for members of the HD-ZIP III family, none of the genes known to regulate cambium stem cell activity have been reported as miRNA targets (**Supplementary Table S2**). HD-ZIP III genes are targeted by miR165/166, which we did not detect as being accumulated. These observations suggest that unidentified miRNA-targeted genes may contribute to the regulation of the cambium. If HWS acts on the phloem-developing side of the cambium, one possibility is that expanded miRNA activity promotes proliferation of additional TA-like cells. Alternatively, central stem cells might divide more actively in the mutants. Taken together with the findings of Kim et al. (2016), our observations are consistent with the existence of TA-like cells in the cambium that contribute to cell division. However, they cannot be taken as direct evidence for the existence of such cells. In our kinetic analyses, the reduced xylem size observed in *hws-1* pedicels was attributable to a shortened expansion phase rather than alterations in division rates. The absence of *HWS* expression on the xylem side, as reported by Kim et al., 2016, is consistent with the lack of division rate changes in xylem-side cells.

We also observed a sharp transition between the early and expansion phases of pedicel growth, suggesting the involvement of a discrete signal that triggers radial expansion. Pedicel growth is tightly linked to fruit development. In the absence of fertilization, pedicel growth fails to initiate, and vascular radial growth does not occur in either wild-type or *hws* mutants (data not shown). In our kinetics experiments, the rapid growth phase initiated approximately two days earlier in *hws* mutants, corresponding to previously reported shifts in ovary development (Damayanti et al., 2019). This suggests that precocious hormonal signals associated with early fruit development in *hws* mutants may underlie the observed shift in growth kinetics. It remains unclear whether a similar shift occurs in central stems. Regardless of these differences, the phloem enlargement ratio in fully developed stems and pedicels of *hws-1* remains nearly identical. Future studies should investigate the relationship between hormonal cues and miRNA production in vascular cells. Such analyses will likely require single-cell miRNA isolation to achieve sufficient resolution.

## Data Availability

The datasets presented in this study can be found in online repositories. The names of the repository/repositories and accession number(s) can be found below: https://www.ncbi.nlm.nih.gov/geo/, GSE299885.

## Supplementary material

Supplementary Table 1 Cross-sectional outer phloem areas in 20-DAA pedicels of WT, *hws-1* and *hws-3*. Areas were not significantly different from a Kruskal-Wallis test, *p* = 0.94. Sample size: *n* = 19, 16 and 5 for WT, *hws-1* and *hws-3*, respectively.

Supplementary Table 2 Key regulatory genes of cambium development.

Supplementary Figure 1 Comparison of phenotype severity between *hws-1* and *hws-3*.
