# Supplementary material for: miRNA accumulation correlates with increased phloem cell proliferation in tomato hawaiian skirt mutants
Source: Front Plant Sci. 2025 Sep 16;16:1649913. doi: 10.3389/fpls.2025.1649913 (PMC12479557; doi:10.3389/fpls.2025.1649913)
Supplement: Supplementary Table 1 — Cross-sectional outer phloem areas in 20-DAA pedicels of WT, hws-1 and hws-3. Areas were not significantly different from a Kruskal-Wallis test, p = 0.94. Sample size: n = 19, 16 and 5 for WT, hws-1 and hws-3, respectively. [file Table1.pdf]

Table S1: Cross-sectional outer phloem areas in 20-DAA pedicels of WT, *hws-1* and *hws-3*. Areas were not significantly different from a Kruskal-Wallis test, $p = 0.94$. Sample size: $n = 19$, 16 and 5 for WT, *hws-1* and *hws-3*, respectively.

**line**	**mean area** (mm^2)	**SD**
WT	0.13	0.04
hws-1	0.12	0.03
hws-3	0.13	0.02
