# Supplementary material for: miRNA accumulation correlates with increased phloem cell proliferation in tomato hawaiian skirt mutants
Source: Front Plant Sci. 2025 Sep 16;16:1649913. doi: 10.3389/fpls.2025.1649913 (PMC12479557; doi:10.3389/fpls.2025.1649913)
Supplement: Supplementary Table 2 — Key regulatory genes of cambium development. [file Table2.pdf]

Table S2: Key regulatory genes of cambium development.
Putative and demonstrated tomato gene orthologs of major *A. thaliana* genes associated with cambium size or differentiation are listed.

family	locus	*At* ortholog	orthology inferred from
	Solyc12g017370	*APL*	(Khedia et al., 2025)
	Solyc08g067180	*JULGI*	(Cho et al., 2018; Nam et al., 2022)
	Solyc07g018070	*SMXL5*	(Cho et al., 2018)
CLE peptides	*Solyc11g066120 (CLE14)*	*CLE41/44*	(Zhang et al., 2014)
	Solyc05g053630 (CLE8)	*CLE42*	
	Solyc09g061410 (CLE12)		
	Solyc09g091810 (CLE13)		
	Solyc05g053640 (CLE33)		
phloem-DOFs (PEARs)	*Solyc02g090310 (Dof10)*	*DOF1.1/OBP2*	(Cai et al., 2013)
	Solyc11g066050 (Dof32)	*DOF2.2*	
	Solyc00g024680 (Dof34)	*DOF2.4/PEAR1*	
	Solyc08g008500 (Dof26)	*DOF3.2/DOF6*	
	Solyc09g010680 (Dof28)	*DOF5.1/PEAR2*	
	Solyc06g075370 (Dof24)	*DOF5.3/TMO6*	
	Solyc06g071480 (Dof23)	*DOF5.6*	
	Solyc04g077490	*ANT*	(Ruiu et al., 2015)
	Solyc04g078650	*WOX4*	(Thomas et al., 2022)
	Solyc05g051640	*PXY*	(Khedia et al., 2025; Thomas et al., 2022)
	Solyc03g093330		
	Solyc04g081240	*ARF5/MP*	(Zouine et al., 2014)
HD-Zip IIIs	*Solyc08g066500*	*HB8*	(Hu et al., 2014)
	Solyc03g012910	*HB15*	
	Solyc02g024070	*PHB*	
	Solyc08g066500	*PHV*	
	Solyc11g069460	*REV*	

References

Cai, X., Zhang, Y., Zhang, C., Zhang, T., Hu, T., Ye, J., Zhang, J., Wang, T., Li, H., and Ye, Z., 2013. Genome-wide Analysis of Plant-specific Dof Transcription Factor Family in Tomato. *Journal of integrative plant biology* [Online], 55(6), pp.552–566. Available from: `https://doi.org/10.1111/jipb.12043` [Accessed May 18, 2025].

Cho, H., Cho, H.S., Nam, H., Jo, H., Yoon, J., Park, C., Dang, T.V.T., Kim, E., Jeong, J., Park, S., Wallner, E.-S., Youn, H., Park, J., Jeon, J., Ryu, H., Greb, T., Choi, K., Lee, Y., Jang, S.K., Ban, C., and Hwang, I., 2018. Translational control of phloem development by RNA G-quadruplex–JULGI determines plant sink strength. *Nature plants* [Online], 4(6), pp.376–390. Available from: `https://doi.org/10.1038/s41477-018-0157-2` [Accessed December 5, 2023].

Hu, G., Fan, J., Xian, Z., Huang, W., Lin, D., and Li, Z., 2014. Overexpression of SlREV alters the development of the flower pedicel abscission zone and fruit formation in tomato. *Plant science*

[Online], 229, pp.86–95. Available from: https://doi.org/10.1016/j.plantsci.2014.08.010 [Accessed May 17, 2025].

Khedia, J., Vishwakarma, A.P., Galsurker, O., Corem, S., Gupta, S.K., and Arazi, T., 2025. Tomato HAIRY MERISTEM4, expressed in the phloem, is required for proper shoot and fruit development. *Horticulture research* [Online], 12(3), uhae325. Available from: https://doi.org/10.1093/hr/uhae325 [Accessed April 23, 2025].

Nam, H., Gupta, A., Nam, H., Lee, S., Cho, H.S., Park, C., Park, S., Park, S.J., and Hwang, I., 2022. JULGI-mediated increment in phloem transport capacity relates to fruit yield in tomato. *Plant biotechnology journal* [Online], 20(8), pp.1533–1545. Available from: https://doi.org/10.1111/pbi.13831 [Accessed September 15, 2022].

Ruiu, F., Picarella, M.E., Imanishi, S., and Mazzucato, A., 2015. A transcriptomic approach to identify regulatory genes involved in fruit set of wild-type and parthenocarpic tomato genotypes. *Plant molecular biology* [Online], 89(3), pp.263–278. Available from: https://doi.org/10.1007/s11103-015-0367-1 [Accessed May 14, 2025].

Thomas, H., Van den Broeck, L., Spurney, R., Sozzani, R., and Frank, M., 2022. Gene regulatory networks for compatible versus incompatible grafts identify a role for SlWOX4 during junction formation. *The plant cell* [Online], 34(1), pp.535–556. Available from: https://doi.org/10.1093/plcell/koab246 [Accessed August 23, 2022].

Zhang, Y., Yang, S., Song, Y., and Wang, J., 2014. Genome-wide characterization, expression and functional analysis of CLV3/ESR gene family in tomato. *Bmc genomics* [Online], 15(1), p.827. Available from: https://doi.org/10.1186/1471-2164-15-827 [Accessed December 14, 2023].

Zouine, M., Fu, Y., Chateigner-Boutin, A.-L., Mila, I., Frasse, P., Wang, H., Audran, C., Roustan, J.-P., and Bouzayen, M., 2014. Characterization of the Tomato ARF Gene Family Uncovers a Multi-Levels Post-Transcriptional Regulation Including Alternative Splicing. Ed. by S. Maas. *Plos one* [Online], 9(1), e84203. PMID: 24427281. Available from: https://doi.org/10.1371/journal.pone.0084203.
