# Supplementary material for: miRNA accumulation correlates with increased phloem cell proliferation in tomato hawaiian skirt mutants
Source: Front Plant Sci. 2025 Sep 16;16:1649913. doi: 10.3389/fpls.2025.1649913 (PMC12479557; doi:10.3389/fpls.2025.1649913)
Supplement: Supplementary Figure 1 — Comparison of phenotype severity between hws-1 and hws-3. [file Presentation1.pdf]

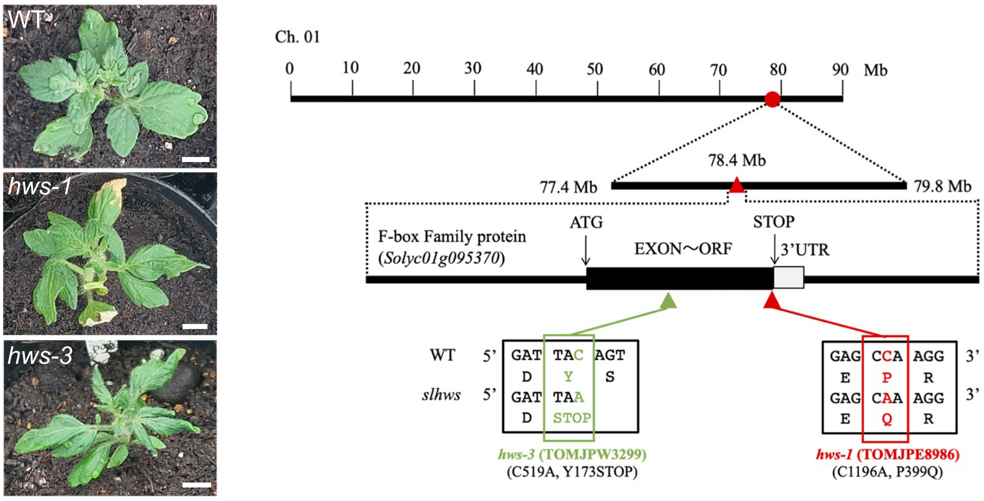

Figure S1: Comparison of phenotype severity between *hws-1* and *hws-3*.
Left: one-month-old plants showing increasing leaf deformations across lines. Bars indicate 1 cm. Right: positions of the mutations in *hws-1* and *hws-3*; modified from **Damayanti2019**.
